# Association between waist-hip ratio and subclinical myocardial injury in the general population: Insights from the NHANES

**DOI:** 10.3389/fendo.2022.975327

**Published:** 2022-09-23

**Authors:** Zhenwei Wang, Xu Huang, Jingjie Li, Naifeng Liu, Qin Wei

**Affiliations:** ^1^ Department of Cardiology, Zhongda Hospital, School of Medicine, Southeast University, Nanjing, China; ^2^ Department of Hematology and Oncology, Affiliated Xuchang People’s Hospital of Xinxiang Medical College, Xuchang, China

**Keywords:** obesity, waist-hip ratio, subclinical myocardial injury, cardiac infarction/injury score, NHANES

## Abstract

**Background:**

Although studies have shown that higher waist-hip ratio (WHR) is closely related to higher risk of metabolism-related diseases, the relationship between WHR and subclinical myocardial injury (SC-MI) is unknown. This study was to evaluate the effect of WHR on SC-MI in the general population free from cardiovascular disease.

**Methods:**

The cross-sectional study included 6253 participants without cardiovascular disease (CVD) from the third National Health and Nutrition Examination Survey (NHANES III) for further analysis. Restricted cubic spline, multivariable logistic regression models and subgroup analyses were performed to assess the association between WHR and SC-MI.

**Results:**

The multivariate logistic regression showed that after adjusting for potential confounding factors, participants in the higher quartiles had higher risk of developing SC-MI than those in the first quartile of WHR [Q3, OR (95% CI): 1.523 (1.159, 2.000), P = 0.002; Q4, OR (95% CI): 1.719 (1.279, 2.311), P < 0.001], and this relationship was robust among the participants aged ≥ 50 years, with hypertension and without diabetes. Every 0.1 unit increase in WHR, as a continuous variable, increased the risk of SC-MI by more than 20% [OR (95% CI): 1.233 (1.092, 1.392), P = 0.001]. And restricted cubic spline analysis showed that there was a linear positive correlation between WHR and the risk of SC-MI.

**Conclusions:**

WHR may be an independent risk factor for SC-MI in the general population free from CVD.

## Introduction

Cardiovascular disease (CVD), mainly including ischemic heart disease and stroke, are the leading cause of death and disability worldwide. In the past 30 years, the prevalence of total CVD has almost doubled from 271 million in 1990, reaching 523 million in 2019, and the number of CVD deaths also increased significantly from 12.1 million in 1990 to 18.6 million in 2019 over that period (1). Although the secondary prevention and rehabilitation treatment of CVD are improving day by day, it seems that CVD is still the main cause of disease burden in the world. Therefore, the primary prevention of CVD is particularly important. Generally speaking, subclinical myocardial injury (SC-MI) occurs before CVD. The SC-MI was defined as cardiac infarction/injury score (CIIS) ≥ 10, without clinically evident coronary heart disease and heart failure (2). As an early stage of CVD, SC-MI has been proved to be directly related to the morbidity and mortality of CVD (3–5). In other words, the higher the CIIS, the higher the risk of CVD. Because of the high risk of SC-MI and the heavy burden of CVD, it is urgent to find and intervene the risk factors of SC-MI.

Previous studies have shown that there is a strong correlation between abdominal obesity and cardiovascular metabolic characteristics (6, 7). In addition, some researchers have shown that abdominal obesity is not only one of the risk factors of insulin resistance, metabolic syndrome, diabetes, hypertension, coronary heart disease and heart failure (8–15), but also related to the increase of cardiovascular mortality and all-cause mortality (16–19). However, little attention has been paid to the effect of abdominal obesity on SC-MI. Abdominal obesity can be measured by a simple, cheap and repeatable method, namely anthropometry measurements, which include body mass index (BMI), waist circumference (WC), waist-hip ratio (WHR) and waist-to-height ratio (WHtR). At present, anthropometry measurements such as WC and WHR, which have been shown to be associated with visceral fat, are widely applied in the measurement of abdominal obesity and broadly used in the epidemiological investigation and clinical work (20–22). Although BMI, WC, WHR and WHtR have basically the same influence on the clinical outcomes of CVD, WHR is considered to reflect abdominal obesity more accurately than other anthropometric indexes for individuals with larger and higher body size (23). Individuals with larger body size but without abdominal obesity tend to have higher WC, which is easy to be misdiagnosed as abdominal obesity. Similarly, individuals with higher body size but with abdominal obesity tend to have lower WHtR and are prone to missed diagnosis of abdominal obesity (19). Consequently, we used WHR as an alternative to reflect abdominal obesity in this study. In this context, the purpose of this study was to evaluate the effect of WHR on SC-MI in the general population free from CVD from the third National Health and Nutrition Examination Survey (NHANES III).

## Methods

### Study population

**Figure 1:**
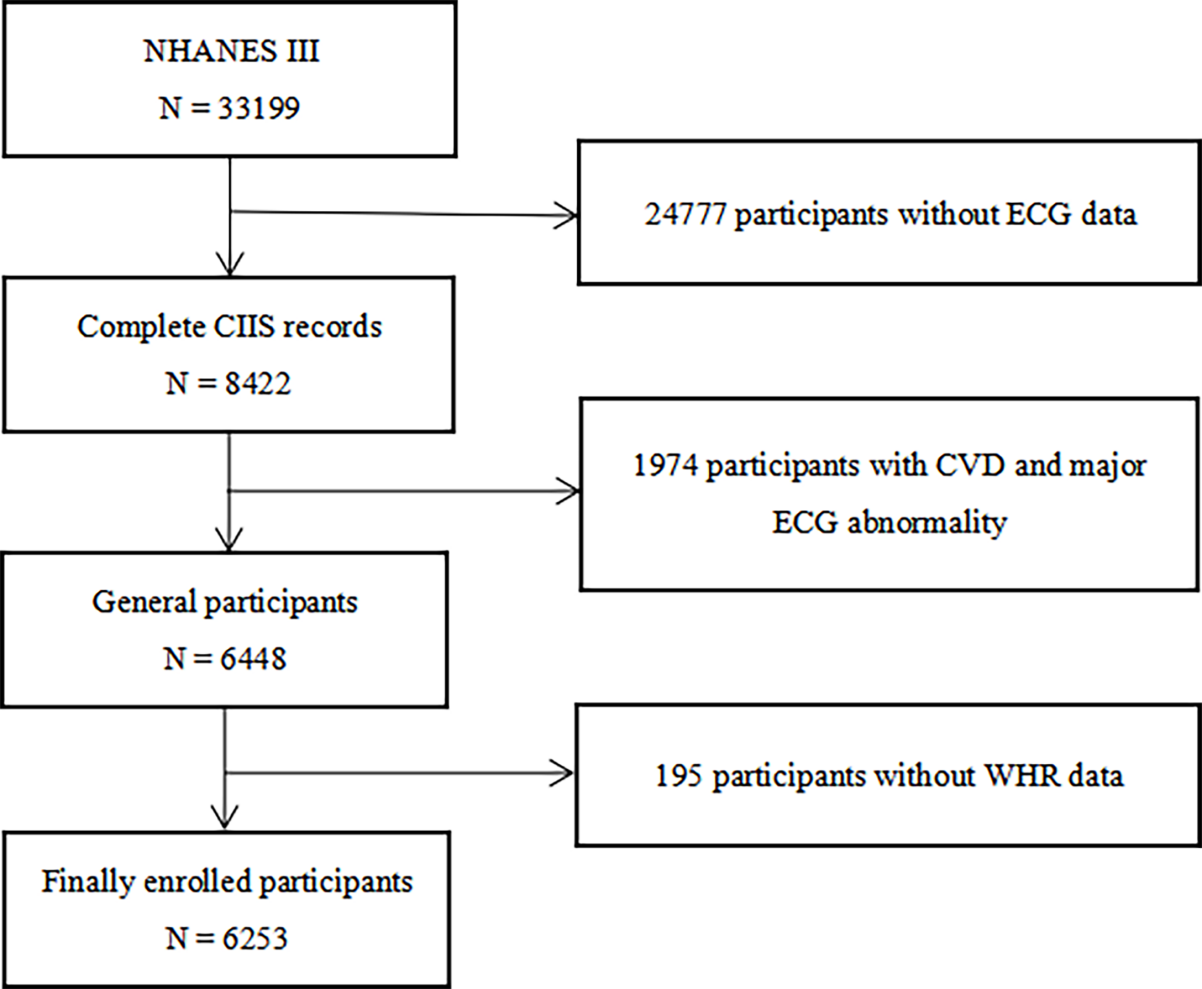
Flow chart of the study population enrollment. NHANES III the third National Health and Nutrition Examination Survey, CIIS cardiac injury/infarction score, CVD cardiovascular disease, ECG electrocardiograph, WHR waist-hip ratio.

All participants were from the NHANES III, which aimed to survey the health and nutrition status of the general population in the United States and offer applicable health guidance, the details and survey data of which have been described in detail elsewhere (24, 25). Participants with CVDs, major electrocardiograph (ECG) abnormality and participants with missing data of ECG and WHR measurements were excluded. Ultimately, 6253 Participants were enrolled in the study (**Figure 1**). The original study protocol was approved by the National Center for Health Statistics of the Center for Disease Control and Prevention Institutional Review Board. Informed consent was waived owing to the retrospective nature of the study, and the study was consistent with the principles of the Declaration of Helsinki.

### Data collection and definitions

The demographic variables of participants, including age, sex, race, history of smoking, hypertension and diabetes, were collected through standardized interview questionnaire. In this study, races were classified into four groups, including non-Hispanic White, non-Hispanic Black, Mexican American and other. Smokers were determined as those who claimed to have smoked more than 100 cigarettes in the past. The anthropometric indexes of each participant, including height, weight, WC, hip circumference (HC), systolic blood pressure (SBP), diastolic blood pressure (DBP), pulse rate, were acquired by the physical examinations. BMI was calculated as weight (kg) divided by the square of the height (meter), expressed as kg/m^2^. WHR was calculated as WC (cm) divided by HC (cm). The blood markers of all participants, including total cholesterol (TC), triglyceride (TG), low-density lipoprotein cholesterol (LDL-C), high-density lipoprotein cholesterol (HDL-C), fasting plasma glucose (FPG), hemoglobin A1c (HbA1c), creatinine, blood urea nitrogen (BUN), uric acid, C-reactive protein (CRP) and fibrinogen, were measured *via* standard biochemistry assays.

The SC-MI was derived from a noninvasive and convenient 12-lead ECG-based risk score, that is, CIIS, which was obtained by adopting a multivariate decision-theoretic ECG classification scheme, and then constructing a risk stratification score system reflecting the severity of myocardial injury by experienced technicians based on the objective ECG waveform components related to myocardial injury or ischemia, more details of which have been described in detail in the original literature (26). As previously mentioned, SC-MI was defined as CIIS ≥ 10, without clinically evident coronary heart disease and heart failure (2, 26).

### Statistical analysis

Continuous variables were expressed as mean ± standard deviation or median (interquartile range), and the one-way ANOVA or Kruskal-Wallis H test was performed to explored the differences between groups depending on the distribution type of the variable. Categorical variables were showed as frequencies (percentages), the Chi-square or Fisher’s exact test was applied to examine the differences between groups. The potential nonlinear correlation between WHR and SC-MI, after adjusting for age, sex, race, smoking, diabetes, hypertension, pulse, SBP, DBP, BMI, TG, TC, HDL-C, LDL-C, fibrinogen, CRP, creatinine, uric acid, BUN, FPG and HbA1c, was assessed using logistic regression models with restricted cubic splines with 3 knots located at the 10th, 50th and 90th percentiles. The multivariable logistic regression analysis was performed to explore the association between WHR and SC-MI in different models with adjustments for covariables with clinical importance and statistical significance (P value < 0.05) in univariate logistic regression analysis. Model 1: unadjusted, Model 2: adjusted for age, sex, Model 3: adjusted for variables included in Model 2 and race, smoking, diabetes, hypertension, and Model 4: adjusted for variables included in Model 3 and pulse, SBP, DBP, BMI, TG, TC, HDL-C, LDL-C, fibrinogen, CRP, creatinine, uric acid, BUN, FPG and HbA1c. Subgroup analysis with multivariable logistic regression stratified by age, sex, hypertension and diabetes was performed to evaluate the association between WHR and SC-MI after adjusting for covariates included in Model 4 when they were not the strata variables. All Statistical analyses were performed using SPSS 19.0 (SPSS Inc., Chicago, Illinois, USA) and R Programming Language (version 3.6.3). A two-tailed P value < 0.05 was regarded as statistically significant.

## Results

### Baseline characteristics of study population

Baseline characteristics of participants were showed in **Table 1**. The study included 6253 participants (mean age: 58.44 ± 13.11 years; 46.20% men). All participants were categorized into four groups based on quartiles of WHR: Q1 (< 0.89), Q2 (0.89 - 0.95), Q3 (0.95 - 1.01), and Q4 (> 1.01). Compared to individuals with lower levels of WHR, participants with higher WHR were older and showed higher percentage of Mexican-American, men, smoker and higher prevalence of hypertension and diabetes (P < 0.001). The levels of BMI, pulse, SBP, TG, TC, LDL-C, fibrinogen, CRP, creatinine, uric acid, BUN, FPG and HbA1c were also higher among population with higher WHR, while level of HDL-C was lower (P < 0.001). Most importantly, participants with higher WHR had higher CIIS and higher prevalence of SC-MI than participants with lower WHR (P < 0.001).

**Table 1:** Baseline characteristics of participants stratified by the quartile of the WHR.

	Total population	Q1	Q2	Q3	Q4	P value
N	6253	1719	1530	1717	1287	
Age, years	58.44 ± 13.11	54.95 ± 12.91	57.63 ± 13.28	59.94 ± 12.84	62.07 ± 12.24	< 0.001
Sex, male, n (%)	2891 (46.2)	209 (12.2)	597 (39.0)	1078 (62.8)	1007 (78.2)	< 0.001
Race, n (%)						< 0.001
Non-Hispanic white	3151 (50.4)	924 (53.8)	736 (48.1)	853 (49.7)	638 (49.6)	
Non-Hispanic black	1392 (22.3)	443 (25.8)	414 (27.1)	349 (20.3)	186 (14.5)	
Mexican-American	1440 (23.0)	261 (15.2)	314 (20.5)	440 (25.6)	425 (33.0)	
Others	270 (4.3)	91 (5.3)	66 (4.3)	75 (4.4)	38 (3.0)	
Smoking, n (%)	3415 (54.6)	740 (43.0)	796 (52.0)	1032 (60.1)	847 (65.8)	< 0.001
Diabetes, n (%)	618 (9.9)	85 (5.0)	128 (8.4)	200 (11.6)	205 (16.0)	< 0.001
Hypertension, n (%)	2021 (32.5)	410 (23.9)	485 (31.8)	617 (36.1)	509 (39.8)	< 0.001
Body mass index, kg/m^2^	27.60± 5.49	26.03 ± 6.07	27.24 ± 5.68	28.02 ± 4.74	29.60 ± 4.60	< 0.001
Pulse, bpm	75.27 ± 12.23	74.38 ± 11.35	75.18 ± 12.41	75.45 ± 12.19	76.32 ± 13.12	< 0.001
SBP, mmHg	131.14 ± 18.95	124.87 ± 18.35	131.10 ± 19.43	134.00 ± 18.41	135.75 ± 17.60	< 0.001
DBP, mmHg	58.11 ± 7.08	58.44 ± 6.59	58.08 ± 7.03	57.93 ± 7.44	57.95 ± 7.30	0.143
Triglycerides, mg/dL	127.0 (90.0, 184.0)	99 (74.0, 142.0)	122.0 (88.0, 168.0)	141.0 (102.0, 202.0)	165.0 (113.0, 241.0)	< 0.001
Total cholesterol, mg/dL	217.75 ± 43.06	212.49 ± 41.82	219.62 ± 43.61	220.93 ± 42.91	218.30 ± 43.63	< 0.001
LDL−C, mg/dL	136.50 ± 38.50	130.04 ± 36.84	138.01 ± 39.60	140.47 ± 38.15	138.25 ± 38.87	< 0.001
HDL−C, mg/dL	51.51 ± 16.47	58.41 ± 16.65	53.43 ± 16.56	48.11 ± 14.97	44.48 ± 13.78	< 0.001
Fibrinogen, mg/dL	309.48 ± 85.12	301.05 ± 78.91	305.83 ± 84.96	312.89 ± 87.01	320.33 ± 89.25	< 0.001
C-reactive protein, mg/dL	0.21 (0.21, 0.50)	0.21 (0.21, 0.40)	0.21 (0.21, 0.44)	0.21 (0.21, 0.50)	0.21 (0.21, 0.60)	< 0.001
Creatinine, mg/dL	1.09 ± 0.35	1.01 ± 0.34	1.07 ± 0.20	1.13 ± 0.32	1.18 ± 0.49	< 0.001
Uric acid, mg/dL	5.40 ± 1.46	4.67 ± 1.26	5.33 ± 1.39	5.69 ± 1.39	6.07 ± 1.44	< 0.001
BUN, mg/dL	15.17 ± 5.52	14.04 ± 5.36	15.02 ± 4.85	15.59 ± 6.00	16.30 ± 5.52	< 0.001
FPG, mg/dL	95.0 (88.0, 105.0)	91.0 (86.0, 99.0)	94.0 (88.0, 103.0)	97.0 (89.0, 107.0)	100.0 (91.0, 114.0)	< 0.001
HbA1c, %	5.74 ± 1.23	5.45 ± 0.88	5.70 ± 1.16	5.84 ± 1.32	6.05 ± 1.46	< 0.001
CIIS	2.2 (0, 8.8)	1.4 (0, 7.3)	1.5 (0, 7.6)	2.6 (0, 9.6)	3.7 (0, 10.7)	< 0.001
SC-MI, n (%)	1342 (21.5)	294 (17.1)	281 (18.4)	407 (23.7)	360 (28.0)	< 0.001

Data are expressed as mean ± SD, median (interquartile range), or n (%).

WHR, waist-hip ratio; SBP, systolic blood pressure; DBP, diastolic blood pressure; LDL-C, low-density lipoprotein cholesterol; HDL-C, high-density lipoprotein cholesterol; BUN, blood urea nitrogen; FPG, fasting plasma glucose; HbA1c, hemoglobin A1c; CIIS, cardiac infarction/injury score; SC-MI, subclinical myocardial injury.

### Association between WHR and SC-MI

**Figure 2:**
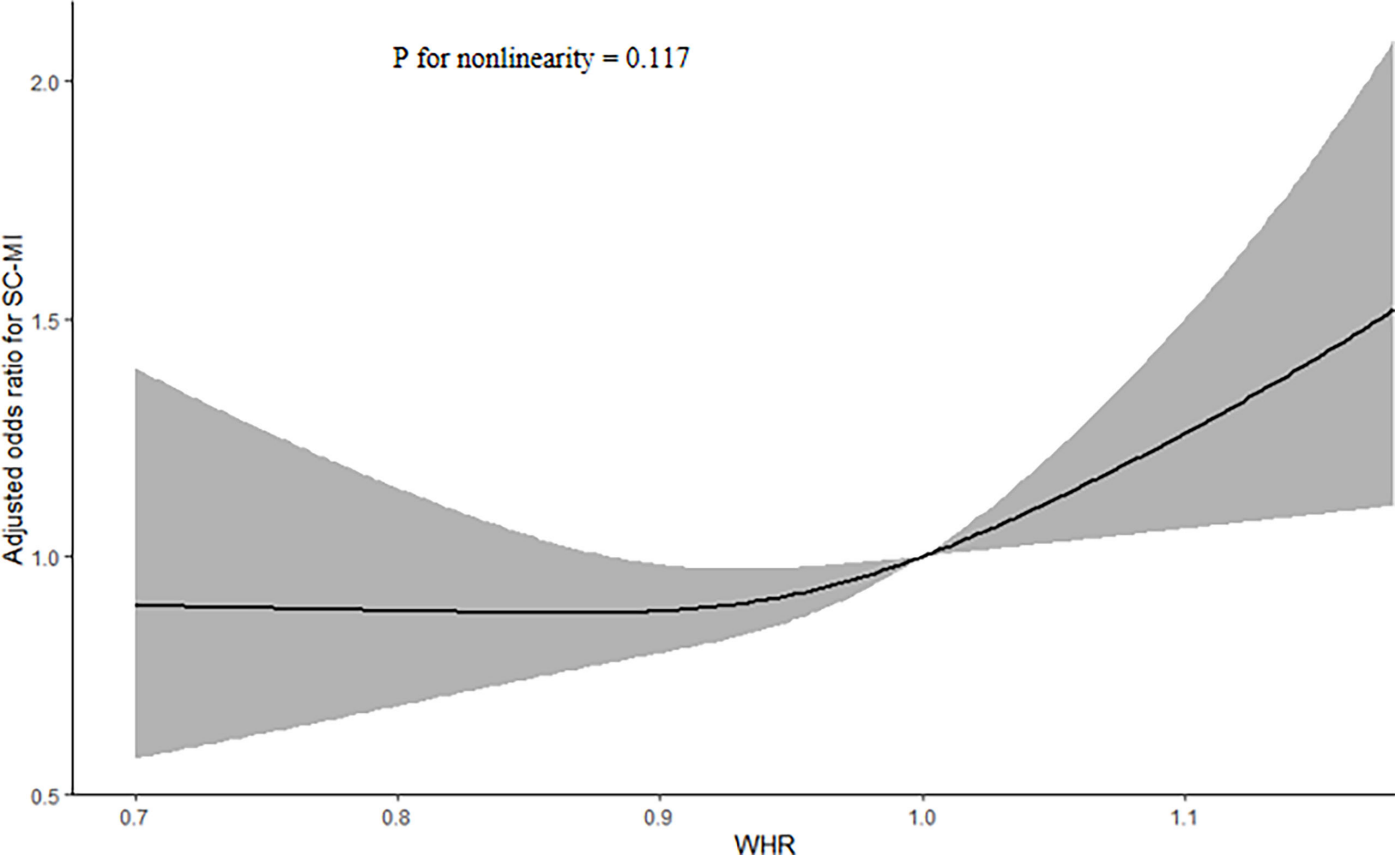
Restricted cubic spline plots of the association between WHR and SC-MI. The association was adjusted for variables included in Model 4. WHR waist-hip ratio, SC-MI subclinical myocardial injury.

The logistic regression analysis results of the correlation between WHR and SC-MI were shown in **Table 2**. When all participants were divided to four groups by quartiles of WHR, population in Q3 and Q4 still had a higher risk of developing SC-MI after gradually adjusting for all potential confounding factors compared with those in Q1 ([Model 1, Q3, OR (95% CI): 1.506 (1.273, 1.781), P < 0.001; Q4, OR (95% CI): 1.882 (1.580, 2.242), P < 0.001]; [Model 2, Q3, OR (95% CI): 1.340 (1.130, 1.589), P = 0.001; Q4, OR (95% CI): 1.599 (1.338, 1.912), P < 0.001]; [Model 3, Q3, OR (95% CI): 1.273 (1.068, 1.517), P = 0.007; Q4, OR (95% CI): 1.487 (1.232, 1.794), P < 0.001]; [Model 4, Q3, OR (95% CI): 1.523 (1.159, 2.000), P = 0.002; Q4, OR (95% CI): 1.719 (1.279, 2.311), P < 0.001]; respectively). When WHR was used as a continuous variable, every 0.1 unit increase in WHR increased the odds of SC-MI by more than 20%, even after adjusting for all potential confounding factors [Model 4, OR (95% CI): 1.233 (1.092, 1.392), P = 0.001]. In addition, restricted cubic spline analysis with full-adjusted Model 4 showed that there was a linear positive correlation between WHR and the risk of SC-MI (P for nonlinearity = 0.117; **Figure 2**).

**Table 2:** Multivariate logistic regression analysis of factors associated with the SC-MI.

	Model 1		Model 2		Model 3		Model 4	
	OR (95% CI)	P value	OR (95% CI)	P value	OR (95% CI)	P value	OR (95% CI)	P value
Q1	Ref	-	Ref	-	Ref	-	Ref	-
Q2	1.090 (0.911, 1.306)	0.346	1.020 (0.850, 1.224)	0.832	0.978 (0.813, 1.177)	0.815	1.018 (0.762, 1.358)	0.906
Q3	1.506 (1.273, 1.781)	< 0.001	1.340 (1.130, 1.589)	0.001	1.273 (1.068, 1.517)	0.007	1.523 (1.159, 2.000)	0.002
Q4	1.882 (1.580, 2.242)	< 0.001	1.599 (1.338, 1.912)	< 0.001	1.487 (1.232, 1.794)	< 0.001	1.719 (1.279, 2.311)	< 0.001
P for trend	-	< 0.001	-	< 0.001	-	< 0.001	-	< 0.001
WHR^a^	1.336 (1.242, 1.438)	< 0.001	1.244 (1.154, 1.341)	< 0.001	1.203 (1.111, 1.303)	< 0.001	1.233 (1.092, 1.392)	0.001

a The OR was examined by per 0.1-unit increase of WHR.

Model 1: unadjusted.

Model 2: adjusted for age, sex.

Model 3: adjusted for variables included in Model 2 and race, smoking, diabetes, hypertension.

Model 4: adjusted for variables included in Model 3 and pulse, systolic blood pressure, diastolic blood pressure, body mass index, triglycerides, total cholesterol, high-density lipoprotein cholesterol, low-density lipoprotein cholesterol, fibrinogen, c-reactive protein, creatinine, uric acid, blood urea nitrogen, fasting plasma glucose, hemoglobin A1c.

WHR waist-hip ratio, SC-MI subclinical myocardial injury, OR odd ratio, CI confidence interval.

### Subgroup analyses

In subgroup analyses based on age, sex, hypertension and diabetes, there was a positive correlation between WHR and the risk of SCMI in almost all subgroups, while this relationship was only statistically significant among the participants aged ≥ 50 years, with hypertension and without diabetes, that is, those with higher WHR were more likely to develop SC-MI in the subgroups with older age, hypertension and without diabetes (P for trend < 0.05). Besides, in the female subgroup, participants in Q3 had a 51.7% risk of developing SC-MI compared to participants in Q1 (Q3 vs Q1: P < 0.05; P for trend = 0.183). Although subgroup analyses demonstrated that WHR and SC-MI had a consistent correlation in age and hypertension subgroups, there was still a significant interaction between WHR and age and hypertension (P for interaction < 0.05) (**Table 3**).

**Table 3:** Subgroups analysis between WHR and the SC-MI.

	Q1	Q2	Q3	Q4		
	OR (95% CI)	OR (95% CI)	OR (95% CI)	OR (95% CI)	P for trend	P for interaction
Age						< 0.001
< 50	Ref	1.057 (0.626, 1.786)	1.588 (0.842, 2.996)	1.824 (0.818, 4.070)	0.331	
**≥ 50**	Ref	1.026 (0.714, 1.474)	1.551 (1.111, 2.166)*	1.677 (1.178, 2.386)**	0.003	
Sex						0.325
Male Female	Ref Ref	1.212 (0.578, 2.545) 1.019 (0.717, 1.450)	1.682 (0.817, 3.465) 1.517 (1.012, 2.274)*	1.796 (0.837, 3.854) 1.242 (0.698, 2.209)	0.256 0.183	
Diabetes						0.272
Yes	Ref	1.038 (0.273, 3.947)	1.093 (0.309, 3.863)	0.608 (0.163, 2.264)	0.583	
**No**	Ref	1.041 (0.772, 1.403)	1.521 (1.145, 2.020)**	1.896 (1.393, 2.580)***	< 0.001	
Hypertension						0.013
Yes**No**	Ref Ref	1.205 (0.715, 2.033) 0.891 (0.615, 1.291)	1.672 (1.025, 2.727)* 1.360 (0.911, 2.031)	2.130 (1.279, 3.547)** 1.345 (0.841, 2.150)	0.013 0.118	

Analyses were adjusted for covariates included in Model 4 when they were not the strata variables. The OR was examined regarding Q1 as reference. *P < 0.05, **P < 0.01, ***P < 0.001. WHR, waist-hip ratio; SC-MI, subclinical myocardial injury; OR, odd ratio; CI, confidence interval.

## Discussion

To our knowledge, this study was the first report on the association between WHR and SC-MI. Our study showed that there was an independent linear and positive correlation between WHR and SC-MI after adjusting for potential confounding factors, including age, sex, race, smoking, diabetes, hypertension, pulse, SBP, DBP, BMI, TG, TC, HDL-C, LDL-C, fibrinogen, CRP, creatinine, uric acid, BUN, FPG and HbA1c. Moreover, we also found that this relationship was more pronounced in population with older age, hypertension and without diabetes.

As an early stage of acute myocardial infarction or ischemic heart disease, SC-MI is often difficult to detect because of its concealed onset, while its risk can not be ignored. Although the improved non-invasive technique can replace or supplement the detection and grading of myocardial injury by ECG, ECG is still the most important tool for evaluating myocardial injury. Minnesota code has become the most widely used ECG classification system in epidemiological study, and its application has significantly improved the degree of standardization of ECG measurement. Therefore, in order to develop a simple tool for evaluating myocardial injury, and to improve the accuracy and stability of ECG measurement, Rautaharju et al. developed CIIS through Minnesota coded ECG classification system (26). By using the combination of 8 binary (single threshold), 3 ternary (two threshold) and 4 ECG features measured on a continuous scale, the most accurate and stable classification is achieved. For the actual visual coding of ECG, the CIIS coding program uses a checklist containing 12 items or 12 ECG features from 12-lead ECG measurements, that is, 5 T-wave amplitude measurements, 4 Q-wave duration measurements or Q-stroke R amplitude ratios, and 3 R or S amplitude measurements (26). And the verification cohort results of CIIS showed that, compared with the conventional ECG diagnostic criteria used in clinic, the diagnostic accuracy of myocardial infarction or injury can be significantly improved by optimizing features and threshold selection and multivariate analysis of CIIS. And the sensitivity and specificity of myocardial infarction detection are 85% and 95%, respectively (26). In most studies, SC-MI is defined as CIIS ≥ 10. Although the CIIS score was evaluated by non-invasive routine 12-lead ECG, its practicability has been confirmed by many studies. And several previous studies have shown that SC-MI or CIIS as an independent high risk factor of coronary heart disease was closely related to cardiovascular mortality and all-cause mortality (3–5). For example, Dekker JM et al. first found that regardless of gender, CIIS, as an ECG-based risk score reflecting SC-MI, was independently positively correlated with coronary heart disease and overall cardiovascular mortality (3). Furthermore, O’Neal WT et al. subsequently discovered that after excluding participants with CVDs and obvious ECG abnormalities and adjusting for all confounding factors, compared with participants without SC-MI at baseline, the risk of cardiovascular deaths among the participants with SC-MI after 10 to 20 years was as high as 26%, and the risk of all-cause deaths was as high as 42%, and this probability also reached 22-78% in all subgroups based on age, sex and race (4). Later, German C et al. also found that after adjusting for traditional risk factors, participants with SC-MI at baseline had a 36% risk of cardiovascular death during a 14-year median follow-up period compared with participants without SC-MI, and ideal daily physical activity could reduce this risk, while less physical activity significantly increased the risk (5). In addition, another prospective cohort study by Dekker et al. confirmed that higher CIIS was closely associated with coronary heart disease morbidity and mortality in middle-aged and older men without CVD (27). Besides, vanDomburg et al. also found in a prospective cohort study involving 3,395 patients who participated in the ASPECT trial that higher CIIS was closely related to the risk of all-cause mortality and CVD-related mortality, regardless of the follow-up time, and this correlation still existed after excluding participants with major abnormal ECG. To sum up, CIIS is reliable in people without CVD (2). Therefore, it is very important to identify and intervene the pathogenic factors of CIIS or SC-MI so as to reduce the risk of cardiovascular and all-cause death at source. Previous studies have confirmed that some factors were related to SC-MI, such as vitamin D deficiency (28), tobacco exposure (29), insulin resistance (30), hemoglobin glycation index (31) and so on. However, there are still some unknown risk factors worthy of further study, for instance, abdominal obesity represented by WHR.

Over the past few decades, the global prevalence of overweight as defined by BMI have been increasing year by year, with the prevalence of overweight among adults rising from nearly 24% of women and 21% of men to about 40% of both sexes, and it was worth noting that the prevalence of obesity had more than doubled from 7% to 16% in women and a crazy fourfold increase in men from 3% to 12% (32). And the close relationship between obesity and the burden of cardiovascular disease has been well known, among which the adverse effects of abdominal obesity are more prominent. In recent years, some studies have shown that WHR was considered as a powerful representative of abdominal obesity because of its stability independent of age and body type (19, 23). Several previous studies have shown that WHR was strongly associated with cardiovascular metabolic characteristics, diabetes, coronary heart disease, cardiovascular mortality and all-cause mortality (15, 19, 33, 34). Among them, Streng et al. observed in a prospective cohort study of 1479 participants that higher WHR was independently associated not only with the prevalence of diabetes and the severity of heart failure, but also with higher mortality risk in women (hazard ratio: 2.23, 95% CI: 1.37-3.63, P < 0.05), but not in male participants, and they also found that WHR was positively correlated with inflammatory markers and mitogen-activated protein kinase cascade in female participants, whereas only positively correlated with platelet activity in men (19). Likewise, in a cohort study of 1396 heart failure participants with left ventricular ejection fraction of 40-49%, Gao et al. showed that no matter how WHR changed over time, it was still significantly positively correlated with the risk of incident CVD, heart failure and all-cause death (hazard ratio: 2.32-4.03), which also highlighted the importance of long-term management of abdominal fat (33). Although the above studies confirmed the correlation between WHR and CVD and the risk of death, it failed to prove the causal relationship. Encouragingly, a recent study of 111986 participants from the UK Biobank showed that each 1-SD increase in WHR, after adjusting BMI by polygenic risk score, was not only related to higher levels of TG, 2-hour glucose and SBP, but also closely related to higher risk of diabetes and coronary heart disease, confirming the causal association of WHR with traditional cardiovascular risk factors and CVD (15). Consistent with the above studies, our study also observed that the higher the WHR, the higher the level of cardiovascular metabolic parameters including glycolipid metabolism and inflammation. Most importantly, there was a close linear positive correlation between WHR and the risk of SC-MI, independent of traditional risk factors including BMI, which could contribute to the early detection, diagnosis and intervention of SC-MI and CVD. Besides, in our study, we found that the relationship between WHR and SC-MI was inconsistent in gender, age, hypertension and diabetes subgroups, that is, the relationship between WHR and SC-MI was more significant in participants ≥ 50 years old, female, with hypertension or without diabetes. However, the conclusion that older age or hypertension is more likely to lead to SC-MI is generally accepted, but the conclusion that the relationship between WHR and SC-MI is more statistically significant in women or non-diabetic participants is relatively novel. First of all, in terms of gender, the gender differences in the incidence, prevalence and mortality of CVD have been fully proved. While in our study, we found that there were also gender differences between WHR and SC-MI, which may also be the highlight of the study or the heterogeneity of the study population. Additionally, there is another possibility. It is well known that in most cases, men are more likely to develop CVD than premenopausal women, so in this study, men may have masked the significant association between WHR and SC-MI, which led to the statistically significant relationship between WHR and SC-MI among female participants. Furthermore, as far as diabetes is concerned, diabetes has been recognized as an independent risk factor for CVD. Participants with diabetes tend to be more likely to develop CVD, including SC-MI. However, due to the high risk of diabetes, this may also weaken the association between WHR and SC-MI in people with diabetes. Therefore, our study found that in the diabetes subgroup analysis, the association between WHR and SC-MI was statistically significant only in the non-diabetic population, which was consistent with the conclusion of the whole population.

Although the above studies have confirmed the relationship between WHR and SC-MI and CVD, the mechanisms were still unclear. After the investigation of the literatures, we found that there may be several underlying mechanisms to mediate the correlation. Firstly, higher WHR tends to reflect more abdominal fat and less hip fat. Previous studies have shown that adipose factors mainly from adipose tissue were associated with a variety of metabolic-related diseases, abdominal fat mainly produced harmful adipokines that led to worse clinical outcomes, while hip fat was the opposite (35–37). In addition, excessive abdominal fat can directly or indirectly cause sympathetic hyperactivity and abnormal secretion of adipose factors including adiponectin and leptin, which in turn leads to the occurrence and development of dyslipidemia, prethrombotic state (such as platelet activation), insulin resistance and chronic inflammation, which are the several established independent risk factors of CVD (19, 38–40). Therefore, the higher the WHR, the higher the risk of CVD, which may be mediated by abnormal fat distribution and adipokine dysfunction. Secondly, several studies have found that WHR was closely related to chronic inflammation and mitogen-activated protein kinase activity (19), and they could cause myocardial remodeling and fibrosis (41), resulting in myocardial injury, and systemic proinflammatory state caused by obesity could lead to inflammation of coronary microvascular endothelial cell, which in turn reduced the bioavailability of nitric oxide and protein kinase G activity, thereby accelerating myocardial injury (39). Thirdly, abdominal obesity tends to indicate higher levels of blood pressure, lipids and glucose and less physical activity, which are closely related to the risks of myocardial injury and CVD (5). Similarly, this study also observed that participants with higher WHR had higher levels of traditional cardiovascular risk factors than those with lower WHR. Furthermore, obesity can easily induce psychosocial mental disorders, such as anxiety, depression and sleep disorders, which successively lead to stress and proinflammatory states, thus increasing the risk of SC-MI in susceptible population (42–44). To sum up, higher WHR may directly or indirectly affect the prevalence of SC-MI by the above mechanisms.

Although the encouraging results obtained in this study are helpful to the management of SC-MI, there are still several limitations. First, as a cross-sectional retrospective study, we failed to determine the causal relationship between WHR and SC-MI. Second, this study did not explore the impact of other obesity markers on the prevalence of SC-MI, nor did it explore which obesity marker was the most effective predictor of SC-MI. Third, WC and HC were measured by different staff in different time and space, so although they have received standardized training, there might still be measurement errors. Finally, this analysis was limited to individuals with NHANES III, and the association between WHR and SC-MI might vary from race to race or pedigree, so the results might not be more widely applicable to other populations.

## Conclusions

In conclusion, our study showed that WHR was an independent risk factor for SC-MI, individuals with higher WHR were more likely to develop SC-MI, which emphasized the importance of the management of abdominal fat in the progression of myocardial injury.

## Data availability statement

The original contributions presented in the study are included in the article/supplementary material. Further inquiries can be directed to the corresponding authors.

## Author contributions

ZW conceived and designed the study. XH and JL were responsible for the management and retrieval of data, contributed to initial data analysis and interpretation. ZW drafted the initial manuscript. NL and QW revised the manuscript. NL and QW were the guarantors of this work and had full access to all the data in the study and take responsibility for its integrity and the accuracy of the data analysis. All authors read and approved the final manuscript.

## Acknowledgments

This work thank the other investigators, the staff, and the participants of the NHANES III for their valuable contributions.

## Conflict of interest

The authors declare that the research was conducted in the absence of any commercial or financial relationships that could be construed as a potential conflict of interest.

## Publisher’s note

All claims expressed in this article are solely those of the authors and do not necessarily represent those of their affiliated organizations, or those of the publisher, the editors and the reviewers. Any product that may be evaluated in this article, or claim that may be made by its manufacturer, is not guaranteed or endorsed by the publisher.
